# Assessment of risky sexual behaviours and associated factors among adolescents in Shewa Robit Town, Northeast, Ethiopia: a cross-sectional study

**DOI:** 10.11604/pamj.2022.41.264.25846

**Published:** 2022-03-31

**Authors:** Mulualem Girma Bizuwork, Awraris Hailu, Mitku Mammo Taderegew, Yonas Girma Bizuwork, Abraham Alebie, Betregiorgis Zegeye

**Affiliations:** 1 Kewot Health Office, Shewa Robit, Ethiopia,; 2 Department of Public Health, Institute of Medicine and Health Science, Debre Berhan University, Debre Berhan, Ethiopia,; 3 Department of Biomedical Sciences, College of Medicine and Health Sciences, Wolkite University, Wolkite, Ethiopia,; 4 USAID HIV Control Grant, Addis Ababa, Ethiopia,; 5 HaSET Maternal and Child Health Research Program, Shewa Robit Field Office, Shewa Robit, Ethiopia

**Keywords:** Adolescent, sexual behaviours, associated factors, Ethiopia

## Abstract

**Introduction:**

adolescents are at high risk of engaging in risky sexual behaviours that could predispose them to several health problems. Cognizant of these facts, studies addressing the root causes for risky sexual behaviours in risk areas like Shewa Robit are mandatory. Therefore, the study was conducted to assess risky sexual behaviours and its determinants in Shewa Robit town, Northeast Ethiopia.

**Methods:**

an institutional-based cross-sectional study was conducted among 349 students using quantitative and qualitative approaches. For the quantitative study, data entry and analysis was done by using Epidata-3.1 and statistical package for social sciences (SPSS)-20, respectively. Binary and multiple logistic regression analysis was used to identify factors associated with risky sexual practices. Odds ratio with 95% confidence interval (CI) was used for measuring the strength of association. Variables with P-value of <0.05 were considered statistically significant. Collected data from the qualitative study was debriefed, categorized, coded and common themes were generated manually. Finally, the findings were triangulated with quantitative findings.

**Results:**

a total of 338 respondents involved in the study. Overall, 168 (49.7%) of respondents have had risky sexual behaviour. Alcohol consumption (Adjusted odd ration (AOR)=5.01, 95%CI; 2.12-11.79), peer pressure (AOR=5.82, 95%CI; 2.97-11.41), grade level (AOR=5.82, 95%CI; 2.06-16.45), and family size (AOR=5.19, 95%CI; 2.24-12.01) were significantly associated with risky sexual behaviour.

**Conclusion:**

risky sexual behaviour in the study area was high. Several families and school-level factors were found to be major determinants of risky sexual behaviours. Public health interventions focusing on adolescent sexual health should be targeted to save those risky adolescents.

## Introduction

Adolescence is the age of transition from childhood to adulthood and accounts for one in six of the world total population. Adolescents are often exposed to a range of risky behaviours [1]. Such risky behaviours are highly linked with sexual abuse, unwanted pregnancies, septic abortions, and substance abuse [2-5]. Evidence shows that nearly 35% of disease burden in the globe has roots in adolescence [6]. According to the World Health Organization (WHO) 2016 reports, more than 1.1 million adolescents aged 10-19 years died annually, mostly from preventable causes related to risky behaviour [1]. Additionally, due to these risky behaviours, there were between 1 million and 4.4 million abortions annually among young women and most of these unsafe abortions are with grave consequences; the great majority in the sub-Saharan African region [7].

Risky sexual behaviour is defined as any sexual activity that may expose an individual to the risk of sexually transmitted infections (STIs) including Human immune deficiency virus (HIV), unplanned pregnancies, unsafe abortion, and psychosocial problems. The extent of risky sexual behaviour doesn´t always limit to risk lifestyle, but it also complemented other risk behaviours such as poor school performance, substance abuse, violence involvement, and impede their health throughout the lifecycles ahead [2, 7, 8]. Studies in Ethiopia still showed that, a considerable number of students had practised risk sexual behaviours. This level ranges from 23% in Arba Minch [9] to 49% in Nekemte [10]. Furthermore, 56.9% and 44.6% of them have not used a condom during the first and last times of sexual intercourse, respectively in Addis Ababa [11].

Cognizant of the above facts, further studies addressing the root causes of risky sexual behaviours among adolescents in risk areas like Shewa Robit are mandatory. Besides, there is no baseline study conducted so far in the study area through divergent study findings reported in different parts of Ethiopia and most of them are quantitative studies. Therefore, this study is aimed to assess risky sexual behaviours and its determinant factors with a mixed approach.

## Methods

**Study setting and design:** a descriptive institution based cross-sectional study was conducted among adolescents attending high school and preparatory school in Shewa Robit town, Northeast of Ethiopia from April 1 to 30/ 2020. Shewa Robit town is located in 220 km Northeast of Addis Ababa, the capital city of Ethiopia. According to the local government authority report, Shewa Robit town has an estimated population of 74, 886 of whom, 39063 were male and 35, 595 females. Its weather condition is very hot and belongs to lowland with longitude and latitude of 10°00' N 39°54´E with an elevation of 1280m above sea level. In the area, adolescents account for 30% of the total figure. A total of 3566 (2046 male and 1520 female) students are enrolled in 2019/2020 fascia year. In terms of infrastructure facilities, there are two primary schools, one secondary school, and one preparatory school.

**Study population and sample size determination:** all adolescents attending high school and preparatory schools in Shewa Robit town were the source population and all selected adolescents attending high school and preparatory schools were the study population. All in-school adolescents attending secondary and preparatory school regularly or at day time were eligible for the study, whereas adolescents who were severely ill during data collection, extension students, and those who were not capable of participating were excluded. The required sample size for the quantitative study was determined using a confidence level of 95%, marginal error 5%, and by considering a 27% proportion from the previous study [8]. Finally, by considering a 15% non-response rate, the final sample size was 349. For the qualitative data, a total of 4 focus group discussions (FGDs) and 6 in-depth interviews have been carried out based on the information saturation level.

**Figure 1:**
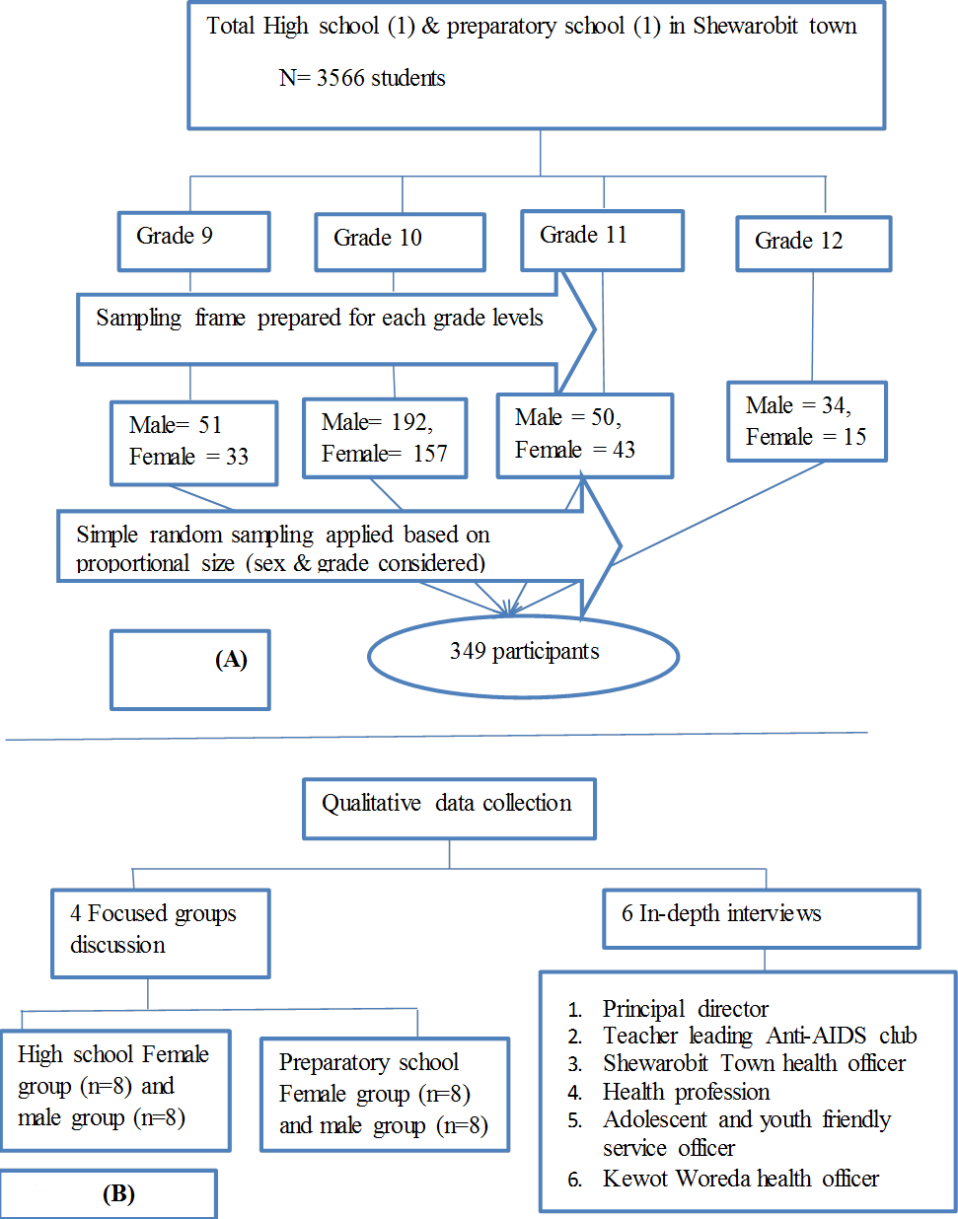
schematic representation of sampling procedures for quantitative and qualitative methods

**Sampling techniques:** for the quantitative method, initially identification of the details of the number of schools, classes, and sections were conducted. The lists of all students (9-12 grades) were prepared from the students´ registration book of each school and grade as a sampling frame. Then, the number of male and female students who were selected from each grade was determined proportionally. Finally, a simple random sampling (lottery) method was used to select each study participants (Figure 1A). For the qualitative method, the study participants were selected through a purposive sampling method by considering their work experience, willingness to take part in the discussion, and accessibility. A total of four FGD and six in-depth interviews have been carried out among teachers/principal directors, students, health administrative officers, and Shewa Robit town administrative women affairs office. The information saturation level ultimately determines whether to end in-depth interviews and FGDs (Figure 1B).

**Data collection procedures:** both quantitative and qualitative data collection methods were employed. For the quantitative part, a pre-tested, self-administered structured questionnaire was used for collecting the data. The tool was adapted from Sexual and Reproductive Health (SRH) questionnaires of the WHO +9 [1]. The questionnaire was originally prepared in English and then was translated to the local language, Amharic, and then back-translated to the English language to check for its consistency. Then the final Amharic form of the questionnaire was used to collect the data. Information on socio-demographic characteristics (age, religion, ethnicity, grade level, family educational status, monthly income of the family, and family marital status), substance abuse (alcohol consumption, chat chewing, and tobacco smoking), sexual behaviours, peer and media related factors were incorporated.

The questionnaire was pretested on 7% of the sample size in Armanya secondary school which is not part of the actual data collection site. Accordingly, all the necessary modification and correction measures were applied. A total of five diploma nurses data collectors and two supervisors (public health professional) were recruited to facilitate data collection. Two-days training was given for data collectors and supervisors by the principal investigator on the objectives of the study, data collection procedure, data collection tools, and confidentiality of information. The principal investigator closely supervised the overall data collection environment and process. Moreover, the collected data were reviewed daily to safeguard its completeness.

For the qualitative part, a semi-structured questionnaire guide or checklist was used. One master of public health (MPH) holder moderator was hired to moderate FGDs. Interviews and FGDs were held in quiet settings. All the discussions and interviews were tape-recorded. At the end of each FGD and interview, the collected information was debriefed, cleaned, and expanded before proceeding to the next. Finally, common themes and sub-themes were synthesized and triangulated with quantitative findings.

### Operational definitions

***Risky sexual behaviour:*** if a student owns at least one of the following: had multiple sexual partners, had sex without a condom with a casual partner, used condom inconsistently with a casual partner in the past 12 months, had sex before the age of 18.

***Consistent condom use:*** using a condom during or at every sexual encounter.

**Data processing and analysis:** the collected data was carefully counted and checked at the end of data collection. Then, it was cleaned, coded, and entered into Epidata version 3.1 and imported into statical package for social sciences (SPSS) version 20 for analysis. Descriptive statistics including frequencies, means, range, and standard deviations were used to summarize the variables. Binary logistic regression analysis was used to assess the association between the outcome and independent variables. All independent variables with a p-value of <0.25 in the unadjusted model of logistic regression analysis were included in the multivariate model. Odds ratio with 95% CI was used for measuring the strength of association. Variables with a P-value of <0.05 was considered as statistically significant.

**Ethics approval and consent to participate:** ethical clearance was obtained from the Ethical Review Board (IRB) Debre Berhan University. Then Permission and supportive letter to carry out the study was also obtained from each school. Before the actual data collection, written informed consent was obtained from the study participants aged 18 years and above. However, some study participants were children (under 18 years of age). Thus, written informed consent was taken from their parents through referring the letter to them at home, and also assent was obtained from those participants age less than 18 years. Detail clarification about the purpose and advantage of the study was explained to the study participant to safeguard their full collaboration. Confidentiality of collected information from each study participant was ensured throughout the study. This is an original study that has not been published before and that is not currently under consideration by any other journal.

## Results

**Socio-demographic characteristics of the study participants:** a total of 349 students were invited and 338 of them were volunteered to participate in the study, yielding the response rate 96.8%. The mean age of respondents was 16.38 (±1.27) years. More than half (55.6%) of the respondents were male. The majority (86.4%) of respondents were Amhara by ethnicity and two hundred twenty-six (66.9%) were Orthodox Christian followers. Regarding marital status, almost all (97%) were single. The family size of respondents ranged from 3 to 15 with a mean size of 7.71 (±1.97) (Table 1). A total of 37 and six participants whose ages ranged from 18 to 40 years old were involved in FGDs or in-depth interviews, respectively. The majority of the FGD participants had educational level at least high school.

**Table 1:** socio-demographic characteristics of the respondents in Shewa Robit town, Northeast Ethiopia, 2020 (n= 338)

Variables	Categories	Frequency	Percent
Sex	Male	188	55.6
	Female	150	44.4
Religion	Orthodox	226	66.9
	Muslim	74	21.9
	Protestant	38	11.2
Age of respondent (years)	13 to 16	184	54.4
	17 to 19	154	45.6
Age at first sex (years)	Below 18	66	39.8
	18 and above	100	60.2
Family size	Below 5	129	38.2
	5 to 7	102	30.2
	Above 7	107	31.6
Ethnicity	Amahara	292	86.4
	Others*	46	13.6
Marital	Single	328	97.0
	Married	10	3.0
Grade level	Grade 9	137	40.5
	Grade 10	85	25.2
	Grade 11	68	20.1
	Grade 12	48	14.2
Perceived economic status related to neighbor	Poor	85	25.1
	Medium	178	52.7
	Rich	75	22.2
Living arrangement	With father and mother	132	39.0
	With father or mother	105	31.1
	With relatives	70	20.7
	Alone	31	9.2

Note: Others* include Argoba, Oromo, and Tigre

**Figure 2:**
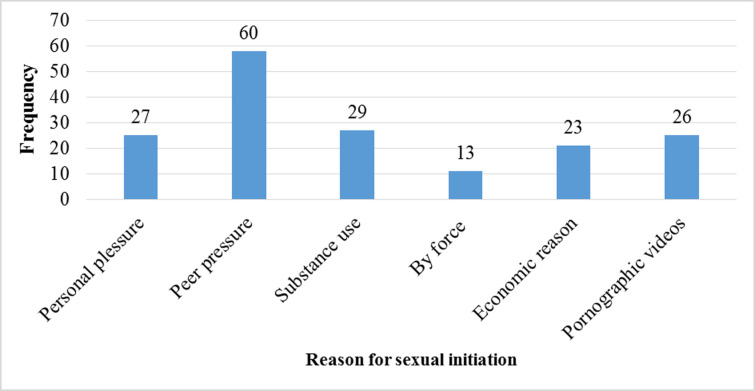
reason for sexual initiation among the study participants

**Sexual behaviours of respondents:** more than half (52.7%) of the students had ever engaged in sexual intercourse. Of whom, 95 (53.4%) were males while 83 (46.6%) were females. The mean age at first sexual engagement was 15.65 (±0.95) years old. Nearly half (49.4%) of sexually active adolescents were below 18 years at the time of sexual initiation. Among sexually active respondents, 59 (33.1%) of them have had more than one sexual partner in the past 12 months. Main reasons for having multiple sexual partners were perceiving all partners are health 34 (57.6%), to have better sexual experience 14 (23.7%), and the difference in sexual pleasure 11 (18.7%). One hundred eighteen (34.9%) respondents have had sex without a condom in the past 12 months. Overall, about 168 (49.7%) of respondents had practised risky sexual behaviours. Two hundred seventy-five (81.4%) of respondents believed that there are no adequate sexual and reproductive health programs in the study area. Peer pressure 60 (33.7%) and the influence of substance use 29 (16.3%) were among the main reasons for the initiation of sexual engagement (Figure 2).

The majority of the key informants and FGD participants have a similar opinion that sexual practice among adolescent students was very high. However, concerning sexual initiation, some supported that students start sexual intercourse before coming to high schools; whereas, most of them argued that most of the students start sexual practice after coming to high school and more pronounced among preparatory students because of lack of parental control, feeling of independence, and self-confidence for their action. Most of the respondents also explained that having multiple sexual partners is very common among students. As they explained, the students need different benefits, including academic as well as economic.

**Factors Associated with risky sexual behaviours:** bivariate analysis revealed that significant factors associated with adolescent risky sexual behaviours were respondent grade level, family size, perceived economic status, attending religious institution/programs, parental monitoring, alcohol consumption, chewing chat, smoking cigarette, peer pressure, watching pornographic videos, age of respondent, living arrangement and having information about sexuality. However, upon multivariate logistic regression, predictors of risky sexual behaviours remained: respondent grade level, alcohol consumption, peer pressure, and family size in the household (Table 2).

**Table 2:** logistic regression analysis of factors associated with risky sexual behaviours among the study participants in Shewa Robit Town, 2020 (n=338)

Variable	Category	Risky sexual behavior		COR (95% CI)	AOR (95% CI)
		No (%)	Yes (%)		
Alcohol consumption	No	142 (74.0)	50 (26.0)	1	1
	Yes	28 (19.2)	118 (80.8)	11.97 (6.91-19.63)*	5.01 (2.12-11.79)*
Peer pressure	No	136 (74.7)	46 (25.3)	1	1
	Yes	34 (21.8)	122 (78.2)	10.61 (6.22-17.07)*	5.82 (2.97-11.41)*
Parental monitoring	Low	40 (33.3)	80 (66.7)	1	1
	Medium	56 (63.2)	31 (36.8)	2.59 (1.55-4.33)*	1.11 (0.49-2.52)
	High	74 (56.5)	57 (43.5)	0.75 (0.43-1.31)	0.42 (0.17-1.04)
Living with	Father and mother	79 (59.8)	53 (40.2)	1	1
	Father or mother	48 (45.7)	57 (54.2)	1.77 (1.09-3.09)*	1.45 (0.67-3.15)
	Relatives	29 (41.4)	41 (58.6)	2.10 (1.16-3.79)*	1.32 (0.54-3.23)
	Alone	14 (45.2)	17 (54.8)	1.81 (0.82-3.98)	0.54 (0.14-2.01)
Information on sexuality	No	36 (66.7)	18 (33.3)	1	1
	Yes	134 (47.2)	150 (52.8)	2.27 (1.23-4.18)*	1.36 (0.54-3.39)
Cigarette smoking	No	134 (65.7)	70 (34.3)	1	1
	Yes	36 (26.9)	98 (73.1)	5.21 (3.16-8.22)**	1.28 (0.59-2.79)
Grade	Grade 9^th^	46 (68.7)	21 (31.3)	1	1
	Grade 10^th^	35 (87.5)	5 (12.5)	0.31 (0.10, 0.91)*	0.53 (0.14-2.05)
	Grade 11^th^	74 (46.8)	84 (53.2)	2.48 (1.36, 4.54)*	1.47 (0.64-3.38)
	Grade 12^th^	15 (20.5)	58 (79.5)	8.47 (4.23-20.10)*	5.82 (2.06-16.45)*
Chewing chat	No	112 (64.7)	61 (35.3)	1	1
	Yes	58 (35.2)	107 (64.8)	3.39 (2.22-5.44)*	1.11 (0.50-2.44)
Watching porn video	No	163 (57.6)	120 (42.4)	1	1
	Yes	7 (12.7)	48 (87.3)	9.31 (4.60-26.74)*	3.40 (0.87-13.21)
Family size	≤5	88 (68.2)	41 (31.8)	1	1
	6-7	61 (59.8)	41 (40.2)	1.44 (0.83-2.48)	0.81 (0.37-1.76)
	>7	21 (19.6)	86 (80.4)	8.79 (5.06-17.20)*	5.19 (2.24-12.01)*
Respondent's age	13-16	116 (63.0)	68 (37.0)	1	1
	17-19	54 (35.1)	100 (64.9)	3.16 (1.97-4.82)*	1.60 (0.81-3.14)
Economic status	Poor	56 (47.9)	61 (52.1)	2.19 (1.23-3.92)*	0.63 (0.27-1.46)
	Middle	59 (49.6)	60 (50.4)	1.01 (0.60-1.70)	1.44 (0.64-3.23)
	Rich	55 (54.5)	46 (45.5)	1	1

N.B: *Significant at p-value <0.05

The odd of students under peer pressure or influence were approximately six times more likely in engaging in risky sexual behaviour (AOR=5.82, 95% CI; 2.97-11.41) than students who had no peer pressure or peer influence. Peer influence was cited by all the participants as the most important factor that influenced students´ sexual risk-taking behaviour. The respondents suggested that their behaviour was a means of gaining entry and certification into a social peer group. Part of being accepted by peers was to emulate their behaviour; in this instance, sexual behaviour was perceived as an official group membership “stamp”.

*“The greater influence is friends because it´s like when your friends tell you I have done that, you also want to try it. We trust our friends so much in things that we shouldn´t even trust them with. Especially here in our schools, people make you feel like you are abnormal if you are not having sex, for some reason. Unfortunately, being a virgin now a day is treated as an insult.”* (19 years old, female prep group).

There was consensus among the interviewees that seeking advice from their peers regarding sexual behaviour was easier than going to an adult. They found it stress-free to converse confidently with friends of the same age group. The respondents highlighted culture and religion as a barrier to talking about sex and sexuality with older health practitioners and older community members. They further expressed fear of being judged by adults when seeking health advice, therefore relied on friends for such. The odd of respondents with more than seven family sizes in the household were approximately five times more in instigating risky sexual behaviours (AOR=5.19, 95% CI; 2.24, 12.01) than the family size of less than five in the household. The qualitative finding also supported this result.

*“Family socioeconomic status and large family size in the household was also cited in this regard and highlighted as playing a key role in risky sexual behaviours. Students from low socioeconomic status and large family size are subjected to higher sexual encounters due to the resource and time constraints of their parents because of their big family size and caring for their children and other house workloads. For that matter, students from such households have limited parental control and may use sex as a source of income generation as well.”* [Health professional, 32 years old].

The odd of students who attended grade 12 were approximately six times more at risk for sexual behaviour compared to grade 9 students (AOR=5.82, 95%CI; 2.06-16.45). This was also supported by the qualitative finding that majority expressed that both high school and preparatory school students are active in our area. Yet, it was more pronounced among preparatory students was leading to engage in sexual intercourse and to have multiple sexual partners as they feel seniority. A 19 years old student stated:

*“…one of my friends had no experience of sexual practices before he came to preparatory, but later on after abstaining for two years, he started sexual practice with high school students.”*

The odd of students who consumed alcohol had five times higher in engaging in risky sexual behaviour compared to those who didn´t consume alcohol (AOR=5.01, 95% CI; 2.12, 11.79). Almost all interviewees spontaneously viewed alcohol as a cornerstone of students´ life and it was viewed as playing a key role in facilitating sexual intercourse. In particular, participants were of the view that the dis-inhibition effect of alcohol and the strategic use of alcohol to facilitate sexual encounters were major contributors to students engaging in risky activities such as unprotected sex.

## Discussion

In this institution-based cross-sectional study, the magnitude of sexually risky behaviour and influencing factors among students has been conducted with a quantitative and qualitative approach. It was found that nearly half of the students were engaged in risky sexual behaviours. It also found that alcohol consumption, peer pressure, student grade level, and family size were the main identified influencing factors for risky sexual behaviour.

Accordingly, the present study revealed that the coverage of adolescent risky sexual behaviours was highest compared to findings from Gondar city, Northwest Ethiopia (12.8%) [8], Arba Minch (23%) [9], East Gojam (26%) [12], Bodity southern Ethiopia (17.9%) [13] and Mizan Aman (31%) [14]. On the other hand, it was relatively consistent with the report in Nekemte (49%) [10], and Nigeria (47.4%) [15]. This could be due to the absence or lack of enabling conditions for adolescents to receive health education on sexuality. About 81.4% of respondents in this study also reported that there are no adequate and comprehensive sexual and reproductive health programs in the area.

Nearly 35% of respondents commenced sex without a condom in the past 12 months. This finding is somewhat comparable to 44.6% of the adolescents who have not used a condom during the last times of sexual intercourse in Addis Ababa [11]. This might be due to their immature unplanned and emotional motives just not to miss opportunities. So, to use their best opportunity, no concern is considered on using a condom. Moreover, they might also feel ashamed and fear to buy a condom from the drug store.

Respondent grade level was found to be one of a significant predictor for risky sexual behaviours. This finding is in line with the study done in Bahirdar [3] and inconsistent with findings from Addis Ababa [8]. This might be due to the study setting and socio-demographic variation. Higher grade levels may also think themselves as senior and at that level substance use getting into nightclubs and engaging in a range of unprotected sex may be misperceived as a sign of classicality or civilization. It is also further explained by the fact that in recent years, there have been extensive social media exposures and the effect of globalization that would increase the unlimited transfer of sexual information´s that in return might lead adolescents towards active sexual involvement and behaviours.

Interviewees further explained that preparatory students are at the stage of transforming into a higher educational institution. As a result, they would like to engage in a variety of friendship moods, declare their independence, and decide a wide range of issues including sexuality by themselves. In this study, respondents who drink alcohol were at higher risk to involve in risky sexual behaviour. As a result, individuals with alcohol influence decide without analysing consequences to be followed after having sex and this could be more pronounced among adolescents. This finding is in agreement with studies in Bahir Dar [3], Gondar city, Northwest Ethiopia [8], Kenya [2], and Brazil [16].

Respondents under peer pressure were more likely engaged in risky sexual behaviour than students who had no peer pressure or influence. These finding is consistent with findings in the Western Zone of Tigray, Northwest Ethiopia [4], Aksum Northern Ethiopia [17], Addis Ababa Ethiopia [17], and Nigeria [7]. This could be due to adolescents spending most of their time with their peers so that peers are the most influential socializing agent for sexuality. Those who had sexually active peers have a great chance of dealing with sexuality on a daily basis. Also, abstinence from sex and focusing on education alone could be considered as a sign of conservative that would subject adolescents to many critics by their peers.

The qualitative finding also adds to this growing literature by highlighting peer pressure as a dominant factor influencing risky sexual decision-making. Students reported a desire to belong to a social best group. In these groups, sexual activity was regarded as a point of entry, thus making it hard for students to negotiate sexual abstinence.

Respondents with more than seven family sizes in the household were also more likely to instigate to risky sexual behaviours than family size of less than five in the household. This could be a family having more than seven children are difficult to track or monitor their child´s behaviour and attitude rather they concern about how to feed them. Furthermore, they devote more emphasis to those under 10 children and the rest could be considered as they are capable of differentiating what is good and bad/right or wrong. Students from such congested family members may be enforced to engage in unprotected sexual intercourse with those who have good economic status and use it as a proven means of income generation.

The strength of the study is that the study used a mixed approach, which made the finding to be a more comprehensive and complete understanding of the factors. The study also has some limitations since it was a school-based study, might not represent adolescents not attended the school. Second, since it was self-reported, the result might be affected by recall bias. And lastly, due to its cross-sectional nature, the causal inference might not be possible.

## Conclusion

This study demonstrated that risky sexual behaviour in the study area was very high. Meantime, respondent grade level, alcohol consumption, peer pressure, and family size in the household were found to be major determinants of risky sexual behaviours. Similarly, considering as modern and feeling as senior, friends influence and become poor are commonly raised reasons for risky sexual behaviour by in-depth interview and FGD participants.

### What is known about this topic

- *Prevalence of risky sexual behaviour in different areas have been assessed;*
- *Predictors for practice of risky sexual behaviour have been assessed in different areas*.

### What this study adds

- *The study provide data regarding burden of risky sexual behaviours among adolescents in the study area, which was not studied yet;*
- *The study provides predictors of risky sexual behaviour (alcohol consumption, peer pressure, grade level, and family size) in the study area*.

## References

[1] World Health Organization (WHO) Adolescent and young adult health.

[2] Ochieng JA (2013). Risky sexual behavior among adolescents attending public secondary schools in Nairobi, Kenya. A Dissertation In Part Fulfilment For The Award Of The Degree Of Master Of Medicine In Psychiatry, University Of Nairobi.

[3] Amare H, Azage M, Negash M, Getachew A, Desale A, Abebe N (2017). Risky sexual behavior and associated factors among adolescent students in Tana Haik high school, Bahir Dar, Northern Ethiopia. International Journal of HIV/AIDS Prevention, Education, and Behavioural Science.

[4] Dadi AF, Teklu FG (2014). Risky Sexual Behavior and Associated Factors among Grade 9-12 Students in Humera Secondary School, Western Zone of Tigray, NW Ethiopia, 2014. Science Journal of Public Health.

[5] Singh SK (2011). Interface of alcohol and risky sexual behaviour among adolescents and youth in low-income slums of Mumbai, India. Journal of Family Welfare.

[6] Taghizadeh Moghaddam H, Bahreini A, Ajilian Abbasi M, Fazli F, Saeidi M (2016). Adolescence Health: the needs, problems, and attention. International Journal of Pediatrics.

[7] Nnebue CC, Chimah UC, Duru CB, Ilika AL, Lawoyin TO (2016). Determinants of Age at Sexual Initiation among Nigerian Adolescents: A Study of Secondary Schools Students in a Military Barracks in Nigeria. American Journal of Medical Sciences and Medicine.

[8] Kasahun AW, Yitayal M, Girum T, Mohammed B (2017). Risky sexual behavior and associated factors among high school students in Gondar City, Northwest Ethiopia. IJPHS.

[9] Mersha A, Teji K, Darghawth R, Gebretsadik W, Shibiru S, Bante A (2018). Risky sexual behaviors and associated factors among preparatory school students in Arba Minch town, Southern Ethiopia. J Public Health Epidemiol.

[10] Waktole ZD (2019). Sexual behaviors and associated factors among youths in Nekemte town, East Wollega, Oromia, Ethiopia: A cross-sectional study. PloS one.

[11] Gizaw A, Jara D, Ketema K (2014). Risky sexual practice and associated factors among high school adolescents in Addis Ababa, Ethiopia, 2014. Fam Med Med Sci Res.

[12] Kassa GM, Degu G, Yitayew M, Misganaw W, Muche M, Demelash T (2016). Risky Sexual Behaviors and Associated Factors among Jiga High School and Preparatory School Students, Amhara Region, Ethiopia. Int Sch Res Notices.

[13] Mph TN, Mph TL, Abebe L, Getachew S (2020). high school and preparatory school students in Mizan Aman Town, Ethiopia Magnitude of risky sexual behaviors, determinants, and consequences among high school and preparatory school students in Mizan Aman Town, Ethiopia. Journal of Midwifery and Reproductive Health.

[14] Olasode OA (2007). Sexual behavior in adolescents and young people attending a sexually transmitted disease clinic, Ile Ife, Nigeria. Indian Journal of Sexually Transmitted Diseases and AIDS.

[15] Sanchez ZM, Nappo SA, Cruz JI, Carlini EA, Carlini CM, Martins SS (2013). Sexual behavior among high school students in Brazil: alcohol consumption and legal and illegal drug use associated with unprotected sex. Clinics (Sao Paulo).

[16] Girmay A, Mariye T (2019). Risky sexual behavior practice and associated factors among secondary and preparatory school students of Aksum. BMC Res Notes.

[17] Cherie A, Berhane Y (2012). Peer pressure is the prime driver of risky sexual behaviors among school adolescents in Addis Ababa, Ethiopia. World Journal of AIDS.

